# Role of *Rahnella aquatilis* AZO16M2 in Phosphate Solubilization and Ex Vitro Acclimatization of *Musa acuminata* var. Valery

**DOI:** 10.3390/microorganisms11061596

**Published:** 2023-06-16

**Authors:** Daniela Landa-Acuña, Marcia Toro, Ricardo Santos-Mendoza, Doris Zúñiga-Dávila

**Affiliations:** 1 Laboratorio de Ecología Microbiana y Biotecnología, Departamento de Biología, Facultad de Ciencias, Universidad Nacional Agraria La Molina, Lima 15024, Peru; danielanda17@gmail.com (D.L.-A.); rsantosme@gmail.com (R.S.-M.); 2 Centro de Ecología Aplicada, Instituto de Zoología y Ecología Tropical, Facultad de Ciencias, Universidad Central de Venezuela, Caracas 1041-A, Venezuela

**Keywords:** pyrroloquinoline-quinone (PQQ) gene, 16S gene, tricalcium phosphate, organic acids, rock phosphate, plant-growth-promoting rhizobacteria (PGPR)

## Abstract

*Rahnella aquatilis* AZO16M2, was characterized for its phosphate solubilization capacity to improve the establishment and survival of *Musa acuminata* var. Valery seedlings under ex-acclimation. Three phosphorus sources (Rock Phosphate (RF), Ca_3_(PO_4_)_2_ and K_2_HPO_4_) and two types of substrate (sand:vermiculite (1:1) and Premix N°8) were selected. The factorial analysis of variance (*p* < 0.05) showed that *R. aquatilis* AZO16M2 (OQ256130) solubilizes Ca_3_(PO_4_)_2_ in solid medium, with a Solubilization Index (SI) of 3.77 at 28 °C (pH 6.8). In liquid medium, it was observed that *R. aquatilis* produced 29.6 mg/L soluble P (pH 4.4), and synthesized organic acids (oxalic, D-gluconic, 2-ketogluconic and malic), Indole Acetic Acid (IAA) (33.90 ppm) and siderophores (+). Additionally, acid and alkaline phosphatases (2.59 and 2.56 µg pNP/mL/min) were detected. The presence of the pyrroloquinoline-quinone (PQQ) cofactor gene was confirmed. After inoculating AZO16M2 to *M. acuminata* in sand:vermiculite with RF, the chlorophyll content was 42.38 SPAD (Soil Plant Analysis Development). Aerial fresh weight (AFW), aerial dry weight (ADW) and root dry weight (RDW) were superior to the control by 64.15%, 60.53% and 43.48%, respectively. In Premix N°8 with RF and *R. aquatilis*, 8.91% longer roots were obtained, with 35.58% and 18.76% more AFW and RFW compared with the control as well as 94.45 SPAD. With Ca_3_(PO_4_)_2_, values exceeded the control by 14.15% RFW, with 45.45 SPAD. *Rahnella aquatilis* AZO16M2 favored the ex-climatization of *M. acuminata* through improving seedling establishment and survival.

## 1. Introduction

Phosphorus (P) is an essential macronutrient required by all plants [1,2]. It is considered, after nitrogen, as the most critical element for agricultural production, of vital importance in plant growth and development [2,3] because of its role in important biomolecules such as nucleic acids, phospholipids and nucleotides [4,5] as well as photosynthesis and various metabolic processes [6]. Widely distributed in nature, it is affected by soil type, pH, vegetation type, microbial activity, and fertilizer inputs [7]. The forms of P present in the soil can be classified into organic and inorganic phosphorus; however, lack of accessibility is common, because it is found in highly insoluble forms in soils [4], whose availability depends on a progressive insufficiency of its natural sources, relative edaphic scarcity, high retention by the soil matrix, lack of natural replenishment and low mobility compared to that of other minerals [8].

Although many soils have a relatively large reserve of total P (about 0.05% on average), only 0.1% of the total is available to plants [9]. Still, this situation is not entirely improved through the application of P fertilizers, as the soluble phosphorus present in fertilizers easily and rapidly precipitates in insoluble forms with Ca^2+^, Fe^3+^, Al^3+^, Co^2+^ or Zn^2+^ cations, or is adsorbed to calcium carbonates, aluminum oxide, iron oxide and aluminum silicate, depending on the particular soil properties [10,11], such that much of the added P loses effectiveness due to a series of abiotic and biotic biochemical processes such as fixation, sorption and immobilization, in addition to representing an environmental risk due to leaching, runoff and erosion [12].

Meanwhile, plants assimilate phosphorus in soluble inorganic forms, such as monobasic (H_2_PO_4_^−1^) and dibasic (HPO_4_^−2^) ions [13,14,15]. In view of this, research recognizes the benefits of the application of bacterial strains to agricultural crops, which favor the mobilization of P in the soil, in addition to offering an alternative for improving crop productivity [16]. In this context, some microorganisms play a very important role in the biochemistry of the phosphorus cycle in natural and agricultural ecosystems, due to their ability to transform insoluble P into forms that are assimilable by plants (H_2_PO_4_^−1^ and HPO_4_^−2^), either through acidification, chelation, exchange reactions or formation of polymeric substances [17], which facilitates the mobilization of nutrients contained in fertilizers [18], in addition to favoring solubilization or mineralization, which influences three dynamic phenomena of the P cycle: sorption–desorption, dissolution–precipitation and mineralization–immobilization [16,19].

Phosphate solubilizing bacteria (PSB) are free-living microorganisms, which may or may not be associated with plant roots and exert a plant-growth-promoting effect. They also play an important role in plant nutrition through increasing available P [20]. Among the plant-growth-promoting effects (PGPR) of phosphate solubilizing bacteria (PSB), the production of auxins, acetic acid-3-indole-3-acetic acid is considered [21,22]. In addition to the ability to mobilize organic and inorganic phosphates, they are considered the most important in plant nutrition [23,24]. Therefore, studies focused on the isolation, characterization, and evaluation of phosphate solubilizing capacity [25] have been carried out with the aim of understanding the solubilizing mechanism and improving crop yield [26,27]. Such mechanisms are associated with a pH decrease and the release of organic acids [28]; on the other hand, they can solubilize P through chelation activities [29] as well as secretion of enzymes such as phosphatase, phytase and C-P lyase that favor the mineralization of organic phosphorus [30].

Banana is a plant that, during the vegetative and reproductive stages, requires an important input of fertilizers [31]. Although it requires only small amounts of P, it is one of the most used nutrients in fertilization [32]. Among the most used forms of phosphorus in banana crops are monoammonium phosphate (10–12% N and 48–61% P_2_O_5_) and diammonium phosphate (18% N and 46%P_2_O_5_); other sources of phosphorus are the following: triple superphosphate (20% P) and single superphosphate (12–18% P_2_O_5_, 18–21% Ca and 11–12% S). Generally, two applications per year are sufficient [33]. Likewise, the presence and dynamics of P in the soil depend on various factors. That is why in certain countries it has become a crop that is highly dependent on inorganic chemical inputs that are easily assimilated by plants, which creates a risk to the environment due to contamination of water resources, leaching, the susceptibility of crops to diseases [34] and human, plant, soil and environmental health [35]. In addition, the banana plant is susceptible to different types of biotic and abiotic stresses; in such sense, phosphate solubilizing plant-growth-promoting bacteria can exert a beneficial effect, increase nutrient bioavailability and enhance beneficial symbiosis with the host [36].

Agriculture in Peru is one of the most important economic activities in the country. Therefore, for farmers, obtaining better yields represents a challenge in production. This productivity, among other conditions, is limited by the availability of phosphorus in the soil, which leads to the excessive use of fertilizers. The mechanism used by certain plant-growth-promoting bacteria in phosphate solubilization represents a valuable tool that can improve the availability of P in soils. *Rahnella aquatilis* AZO16M2, could improve phosphorus availability under different pH and temperature conditions, through different mechanisms such as the production of organic acids, phosphatases and siderophores, as well as influencing the establishment and survival of *M. acuminata*, “Banana” seedlings, during the first growth phase under ex-acclimation conditions.

## 2. Materials and Methods

### 2.1. Semi-Quantitative Estimation of Phosphate Solubilization Capacity: Effects of Temperature and Initial pH of the Medium

From a pure culture of *R. aquatilis* AZO16M2 previously isolated from potato rhizosphere [37], prepared in nutrient broth at 28 °C for 24 h, 10 µL were inoculated at three equidistant points in Petri dishes with NBRIP solid medium, supplemented with Ca_3_(PO_4_)_2_, AlPO_4_ and FePO_4_ as the sole source of phosphorus. The pH of the medium was previously adjusted to pH 4.5, 5.5, 5.5, 5.5, 6.8, 8 and 9 according to the phosphorus source tested. They were then incubated at temperatures of 4 °C, 6 °C, 28 °C, 35 °C and 40 °C for a period of 18 days. The 0.5% bromocresol green indicator added to the NBRIP medium helped to show the decrease in pH as well as the translucent zones around the inoculation points. The diameter of the solubilization halo (translucent zone around the colony) was measured daily. The effect of temperature and pH was performed on the determination of the solubilization index (SI), according to the formula SI = Halo diameter (mm)/Colony diameter (mm) [38,39] and the determination of the Relative Solubilization Efficiency (RSE), which indicates the range of strain performance in the medium expressed as a percentage in relation to the diameter of the inoculation point: RSE = solubilization halo diameter/colony diameter × 100 [40].

The data obtained were subjected to an analysis of variance (*p* < 0.05) under a complete randomized design (CRD) with factorial arrangement. The factors were type of phosphorus source, temperature with four levels (4 °C, 6 °C, 28 °C, 35 °C) and pH with six levels (4.5, 5.5, 5.5, 5.5, 6.8, 8 and 9). In case of significance, the treatments were compared with Duncan to determine differences between phosphorus source type, pH and temperature.

### 2.2. Quantitative Estimation of Phosphate Solubilizing Capacity

The inoculum was prepared in nutrient broth and incubated at 28 °C for 24 h. Then, 1 mL (1 × 10^7^ CFU/mL) was inoculated in 150 mL of NBRIP liquid medium with Ca_3_(PO_4_)_2_, AlPO_4_ or FeO_4_ as the sole phosphorus source and incubated at 28 °C at 150 rpm. The incubation time was a function of the phosphate solubilizing activity and growth rate of each strain. The quantification of soluble phosphorus was calculated according to the Phosphomolybdenum Blue technique. For this, 5 mL of the bacterial culture was taken every 2 h, centrifuged at 10,000× *g* rpm for 10 min and filtered through 0.22 µm cellulose acetate membranes. The filtrate was then subjected to photometric determination at 690 nm, in triplicate according to the standard method of Murphy and Riley [41]. At the same time, viable cells were counted using the plate incorporation technique and pH measurement.

### 2.3. Determination of Siderophores

Pure cultures in LB medium with 24 h of incubation were used. An amount of 10 µL of the culture was added on plates containing Chrome Azurol Sulfonate Agar (CAS) according to the method of Schwyn and Neilands [42] as detailed in Louden et al. [43], as well as following the detailed preparation of Louden et al. [44]. The plates were incubated for three to five days at 28 °C. The presence of siderophores in the medium was assessed based on the appearance of a color change in the medium surrounding the inoculation point [44,45]. The assay was performed 2 times and in triplicate each time. An additional pure culture was used as a negative control.

### 2.4. Quantification of Acid and Alkaline Phosphatases

Measurement of acid and alkaline phosphatases was performed under the protocol of [46]. It was carried out from the supernatant of NBRIP liquid culture medium with Ca_3_(PO_4_)_2_ inoculated with the pure strains and incubated at 28 °C for 24 h. Once centrifuged at 150 rpm, 100 µL of p-nitrophenyl phosphate (pNPP) solution (0.05 M), 50 µL of MgCl_2_ (0.02M) and 0.1 M universal buffer at pH 6.5 or pH 11, depending on whether for acid or alkaline phosphatase, were added to 50 µL of the supernatant. The samples were then incubated for 1 h at 37 °C, after which the reaction was stopped with 500 µL NaOH (2N), and its absorbance was measured at 410 nm. The amount of phosphatase obtained corresponds to the amount of p-nitrophenol (pNP) released per minute and per ml (µg pNP/mL/min).

### 2.5. Quantification of Indole Acetic Acid (IAA)

For this purpose, LB culture broths supplemented with L-tryptophan (100 mg/L) inoculated with the pure strains were used. The broths were incubated at 28 ± 2 °C in the dark for 48 h. The cultures were then centrifuged at 9500 rpm for 15 min. The supernatants were used for IAA determination via Salkovski’s method [47,48].

### 2.6. Presence of the PQQ Gene Involved in Phosphate Solubilization

Based on the results obtained via high-performance liquid chromatography, which evidences the production of organic acids, we proceeded to verify the presence of the gene encoding the cofactor pyrroquinoline-quinine (PQQ) associated with glucose dehydrogenase (GDH), present in phosphate solubilizing bacteria [23,49]. For this purpose, to verify the identity of the selected strains, genomic DNA was extracted from pure strains, whose 16S rRNA gene sequencing was analyzed with the universal primers rD1 (5′-TAAGGAGGAGGTGATCCAGCC-3′) and 800r (5′-TACCAGGGTATCTAATCC-3′). Likewise, primers were constructed for the recognition of PQQ genes and amplification via the polymerase chain reaction (PCR). The amplified sequences were compared with the GenBank database (https://www.ncbi.nlm.nih.gov/genbank/, accessed on 12 January 2023) through NCBI (http://www.ncbi.nlm.nih.gov/, accessed on 12 January 2023) and the use of the BLAST program (Basic Local Alignment Search Tool, https://blast.ncbi.nlm.nih.gov/Blast.cgi, accessed on 12 January 2023); for verification of alignments, the BioEdit program version 7.0 was used. The evolutionary analysis was performed with the MEGA-X program version 10.0.5. The phylogenetic tree and evolutionary analysis was performed with the program MEGA-X version 10.0.5. The sequences of species of the genus *Rahnella* used as outgroup sequences were obtained from the GenBank database.

### 2.7. Provenance of Clones of Musa Acuminata var. Valery in Nursery

The clones of *M. acuminata* were supplied by the companies Avo Hass Perú S.A.C. and Biotechnology Center S.A.C. and came from in vitro multiplication in temporary immersion systems of 21 days of cultivation. All clones were cultivated and manipulated in sterile conditions. They were then subjected to transplant preparation.

### 2.8. Transplanting, Irrigation and Fertilization

The preparation of *M. acuminata* seedlings from multiplication in temporary immersion systems consisted of initial washing with sterile water, with subsequent removal of incipient roots and necrotic leaves to clear the corm of the seedling to allow direct contact with the substrate and the bacterial inoculum; the whole procedure was carried out in a laminar flow chamber. Then, the seedlings were transplanted in the substrates according to each treatment (sand:vermiculite or Premix N°8), followed by the application of the phosphorous source as background fertilization and finally the bacterial inoculum was placed at the base of the corm of each seedling. They were then covered and fixed with remaining portions of the substrate and placed in the climate chamber until they were ready to be taken to the greenhouse weeks later.

Macronutrients and micronutrients were added using Hoagland and Arnon [50] nutrient solution devoid of P, starting from the second week after planting, according to the needs of the crop: approximately 100 mL per day per seedling. After three weeks, all treatments were placed in the greenhouse.

### 2.9. Statistical Analysis and Experimental Design

For the statistical analysis, the Statistical Package for Social Sciences (SPSS) program of the IBM company version 26 was used. The data obtained in each experiment were subjected to an analysis of variance under a Complete Randomized Design (CRD) with a factorial arrangement, according to the factors tested. The factors tested were substrate factor with two levels (substrate sand:vermiculite, 1:1 [*v*/*v*] and formulated substrate Premix^®^ N°8), phosphorus source factor with two levels (Ca_3_(PO_4_)_2_ and rock phosphate, RP) for the treatments that considered the association with bacteria and three levels for the controls (rock phosphate, Ca_3_(PO_4_)_2_, K_2_HPO_4_) and bacteria factor with the levels established for that moment. An additional absolute control (B0 = No bacteria) and a control without phosphorus source (P0) were added for the tested substrate types. For each treatment, 5 replicates were considered, with a total of 100 experimental units. In all the experimental units with phosphorus source, the equivalent of 31 g of P was applied according to the sources Ca_3_(PO_4_)_2_ and rock phosphate; the amounts were calculated according to their molecular weight and were applied at a single moment in the sowing of the clones. In case of significance, the treatments were compared using Duncan’s test to determine the differences between the bacteria, substrate type and phosphorus source tested. The probability of alpha error of less than 5% was considered significant.

### 2.10. Parameters Evaluated

The parameters evaluated were associated with the growth variables of the seedlings such as plant height, root length, root fresh weight, aerial fresh weight, root dry weight, aerial dry weight, number of leaves, total leaf area and chlorophyll content (SPAD). The measurement of the response variables was carried out in five replicates of each treatment. Leaf area was estimated via a non-destructive method, using a regression equation on an initial sample of 100 leaves belonging to additional units of the experiment, which relates the maximum leaf length and width. According to Rodriguez et al. [51] and Khan et al. [52], leaf chlorophyll content was estimated with the SPAD 502 Plus Chlorophyll Meter. Since the amount of light captured by the SPAD 502 cell is inversely proportional to the amount of light used by the chlorophyll, the equipment processes the signal, and the absorbance is quantified in dimensional values ranging from 0 to 199, so that the SPAD units will be the same according to the green tone of the leaves [53].

## 3. Results

### 3.1. Effect of Temperature and Initial pH in Solid Medium

**Figure 1:**
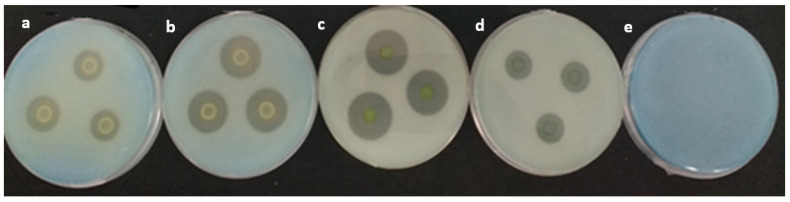
Solubilization halos of *R. aquatilis* (AZO16M2) in NBRIP with Ca_3_(PO_4_)_2_ at different temperatures at pH 6.8. From left to right: temperatures tested (**a**) 4 °C, (**b**) 6 °C, (**c**) 28 °C, (**d**) 37 °C and (**e**) control without bacteria.

**Figure 2:**
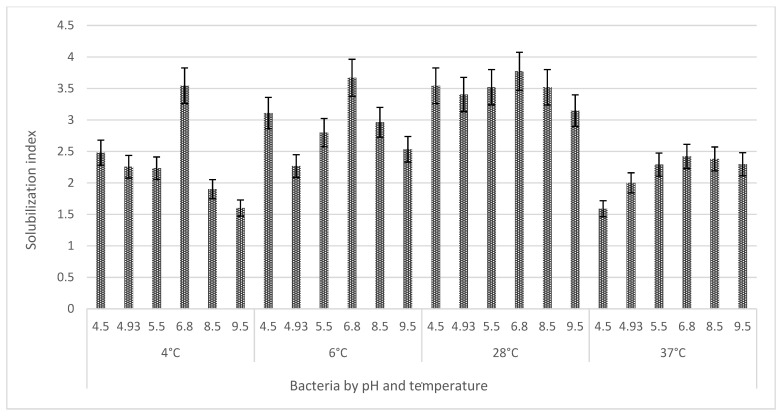
Solubilization index in Ca_3_(PO_4_)_2_ medium under different temperature and pH conditions. The bars correspond to the average of four replicates per treatment and the bars to the standard deviation.

**Figure 3:**
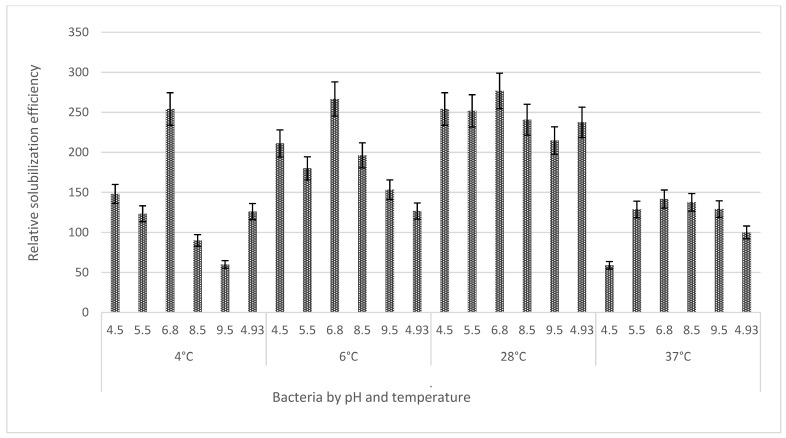
Relative solubilization efficiency in Ca_3_(PO_4_)_2_ medium under different temperature and pH conditions. The bars correspond to the average of four replicates per treatment and the bars to the standard deviation.

The measurement of solubilization halos is part of the preliminary evaluation of the phosphate solubilization capacity [40,54] in solid medium (NBRIP) (Figure 1) through the measurement of the Solubilization Index (SI) and the Relative Solubilization Efficiency (RSE). *R. aquatilis* AZO16M2 only produced solubilization halos in the presence of Ca_3_(PO_4_)_2_, but not in AlPO_4_ and FePO_4_. *R. aquatilis* AZO16M2 generated a maximum SI of 3.77 (28 °C) at pH 6.8. With greater stability at temperatures between 6 °C and 28 °C, at pH 6.8, IS was close to 3.67 and 3.77. Likewise, it was able to solubilize Ca_3_(PO_4_)_2_ in a wide pH range from 4.5 to 9.5, where halo generation was generated (Figure 2). As for the ERS, the maximum efficiencies obtained were 266.67 (6 °C) and 276.85 (28 °C) at pH 6.8 (Figure 3).

### 3.2. Quantification of Available Phosphorus and pH Evolution

Quantification of available P was carried out in NBRIP liquid medium with Ca_3_(PO_4_)_2_, AlPO_4_ and FePO_4_ as the only P source, accompanied by pH measurement and evaluation of population growth of *R. aquatilis* AZO16M2 (Table 1). Cell concentration was associated with the decrease in pH for the phosphorus sources tested (Table 1).

Minimum soluble P values were quantified in the presence of AlPO_4_ and FePO_4_ compared to the medium with Ca_3_(PO_4_)_2_. Meanwhile, the maximum cell concentration was detected in the presence of Ca_3_(PO_4_)_2_, with a slight increase in the presence of FePO_4_ and a gradual decrease without reaching maximum peaks in the presence of AlPO_4_ (Table 1).

### 3.3. Synthesis of Siderophores, Phosphatases, AIA and Organic Acids

**Figure 4:**
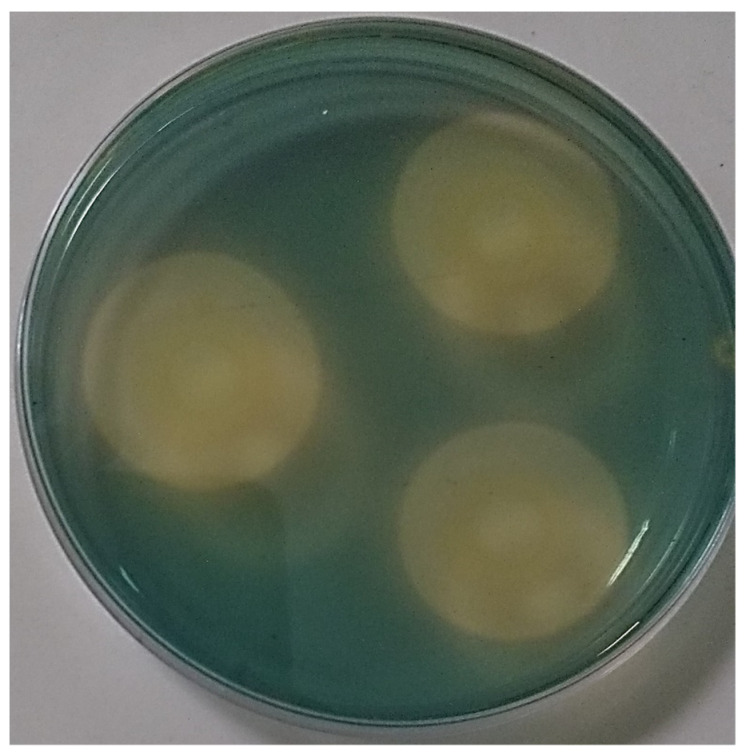
Presence of clear (yellow) halo in CAS medium produced by *R. aquatilis* AZO16M2.

The synthesis capacity of siderophores of *R. aquatilis* was determined through observing the color change from blue to brown around the inoculation point in CAS medium (Figure 4). Likewise, *R. aquatilis* AZO16M2 showed synthesis capacity of organic acids (oxalic acid, D-gluconic acid and 2-ketogluconic acid). Alkaline and acid phosphatases were also demonstrated (Table 2).

The concentration of organic acids synthesized by *R. aquatilis* AZO16M2 varied according to the pH and the temperatures tested (Table 3). Maximum concentrations of oxalic acid were detected at 28 °C, at pH 6.8 and 8.5, while 2-ketogluconic acid registered higher concentrations at 6 °C, 28 °C and 35 °C, at pH 6.8 at each temperature, unlike D-gluconic acid, with presence at 4 °C and 6 °C, at pH 5.5 (Table 3).

### 3.4. Presence of the PQQ Gene Involved in Phosphate Solubilization

**Figure 5:**
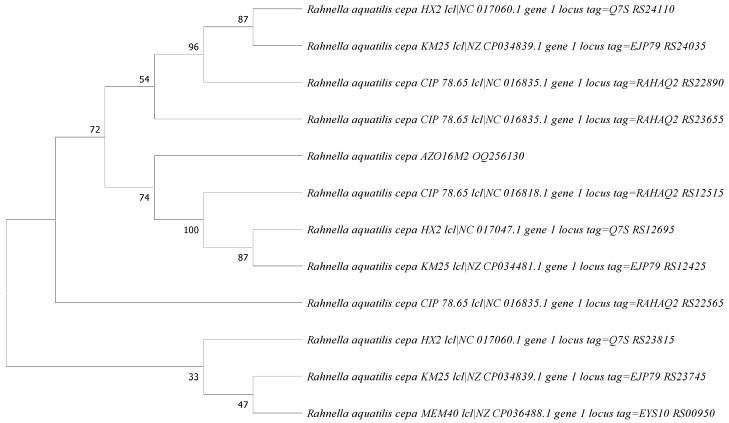
Phylogenetic tree built according to the maximum likelihood statistical method that highlights the relative position of *R. aquatilis* (AZO16M2) in relation to other *Rahnella* species, based on the pyrroquinoline-quinone (PQQ) gene sequence. The sequences of the *Rahnella* species were chosen according to the presence of the PQQ gene as an outgroup.

As part of the study, we proceeded to corroborate the identity of the AZO16M2 bacterium with the use of the 16S rRNA gene. The percentage of similarity with the species described in the NCBI database was obtained and entered with the accession code designation OQ256130. The analysis of the PQQ gene of *R. aquatilis* AZO16M2 was carried out using the neighbor-joining tree test with the statistical method of maximum likelihood, under the Tamura three-parameter substitution model (Gamma distribution 2.82). The consistency of the phylogenetic tree is sustained at 500 Bootstrap replications. Phylogenetic analysis sought to relate *R. aquatilis* AZO16M2 to other *Rahnella* species (outgroups) based on the PQQ gene sequence. The analysis grouped AZO16M2 into a single clade with 75% certainty with the sequences of *Rahnella aquiatilis* CIP, *R. aquatilis* HX2 and *R. aquatilis* KM25 (Figure 5). Likewise, it can be observed that it has a similarity of 72% with other strains of *R. aquatilis* from the neighboring clade. Analysis of the PQQ sequence establishes a phylogenetic relationship between members of the same species that can synthesize organic acids such as gluconic acid.

### 3.5. Response of M. acuminata var. Valery in Association with Phosphate-Solubilizing PGPR Bacteria in a Sand:Vermiculite Substrate

When sand and vermiculite were used as substrate in a 1:1 (*v*/*v*) ratio, a significant interaction was observed between the factor type of bacteria and phosphorus source in the following variables evaluated: For a variable number of leaves, the effect of the bacteria factor was statistically equal between AZO16M2 and the controls, not surpassing the soluble phosphorus source (K_2_HPO_4_) applied. Regarding the phosphorus source factor, a better response was found in the application of RP compared to the application of Ca_3_(PO_4_)_2_, for the variables chlorophyll content, aerial fresh weight, aerial dry weight and root dry weight, surpassing the values obtained by the control (Table 4). On the other hand, no significant differences were observed in the treatments with Ca_3_(PO_4_)_2_ as the only phosphorus source.

The influence of *R. aquatilis* on nutrient uptake by *M*. *acuminata* var. Valery, under ex vitro conditions, is substantially associated with the type of substrate and phosphorus source for some nutrients. Thus, a marked superiority in K% and Mg% is observed with respect to the control with RP, as well as K% with Ca_3_(PO_4_)_2_ in sand:vermiculite substrate. On the other hand, nutrient uptake was not substantial to the type of substrate and phosphorus source for N% and P% with a slight increase in Ca%, K% and Mg% in the presence of Ca_3_(PO_4_)_2_ and rock phosphate, while Ca% did not show changes with respect to the controls (Table 5).

### 3.6. Response of M. acuminata var. Valery in Association with Phosphate-Solubilizing PGPR Bacteria in PREMIX^®^ N°8 Substrate

When using PREMIX^®^ formulated substrate, a significant interaction was found between the factor type of bacteria and phosphorus source in the following characteristics evaluated: root length, aerial fresh weight, root fresh weight and chlorophyll content, when the phosphorus source used was tricalcium phosphate (Table 6).

Regarding the influence of *R. aquatilis* on the uptake of nutrients by *M. acuminata* var. Valery, in ex vitro conditions, it is not substantial, associated with the type of substrate and phosphorus source. Thus, a slight superiority is observed in N%, P% and K% compared to controls with RP in Premix No.8 substrate; a slight increase in Ca%, K% and Mg% is evident in the presence of Ca_3_(PO_4_)_2_ and rock phosphate compared to the controls (Table 7).

The interaction between *R. aquatilis* AZO16M2, the phosphorus source and the type of substrate with respect to the establishment of *M. acuminata* var. Valery seedlings under ex vitro conditions allowed *M. acuminata* var. Valery seedlings to obtain a survival rate of 75%, surpassing the control with Ca_3_(PO_4_)_2_ in sand:vermiculite, whose percentage is similar to the control with K_2_HPO_4_. On the other hand, in Premix N°8 with Ca_3_(PO_4_)_2,_ there was a higher survival rate of 87%, a value that exceeds the controls (Table 8).

## 4. Discussion

The measurement of solubilization halos is part of the preliminary evaluation of the phosphate solubilization capacity [40,54] (Figure 1), carried out through the determination of IS and ERS [55,56,57]. The presence of halos around the colonies of *R. aquatilis* AZO16M2 evidenced the acidification of the culture medium as determinant in phosphate solubilization described in bacteria [22,58,59,60]. The production of organic acids causes the reduction of the pH of the medium [61,62,63,64] and is a determining factor in phosphate solubilization (Table 1), as reported in several bacterial species [65,66]. The synthesis of organic acids is related to the presence of the PPQ gene in some bacteria. According to Behera et al. [23], the main mechanism is the production of gluconic acid from the direct oxidation of glucose, a process that is carried out by glucose dehydrogenase and the cofactor pyrroloquinoline quinone (PQQ). Confirmation of the existence of the PQQ gene in *R. aquatilis* AZO16M2 and sequence analysis phylogenetically links it to members of the same genus *Rahnella* that can synthesize gluconic acid. Moreover, its regulation is a function of the conditions given during microbial growth [67], which confirms the variation of IS and ERS according to the pH and temperature conditions imposed, with maximum IS and ERS detected between 6 °C and 28 °C at pH 6.8 (Figure 2 and Figure 3). Likewise, in liquid medium, the type of organic acid synthesized was a function of the variation of these parameters (pH and temperature) (Table 3). On the other hand, several cloning and gene expression studies have reported the importance of the PQQ gene as a necessary cofactor for gluconic acid synthesis [68,69,70,71].

Differential production of organic acids among bacterial species, attributed to substrate conditions depending on the phosphorus source used [46,65,72,73], has been reported by several researchers [74,75,76]. *R. aquatilis* AZO16M2, in liquid medium with different phosphorus sources, yielded minimum soluble P values when using AlPO_4_ and FePO_4_ compared to Ca_3_(PO_4_)_2_ (29.6 mg/L soluble P) (Table 1). The results obtained from the use of AlPO_4_ and FePO_4_ would not be associated with the reduction of the pH of the medium. However, pH reduction in AlPO_4_ media is caused by the synthesis of organic acids that act as chelating agents, where their ligands or anions form one or more bonds with the Al^3+^ ions of soluble phosphates to form cyclic structures and release phosphates [77]. The results obtained in the present investigation suggest that the drastic reduction in pH is caused by AlPO_4_ and FePO_4_ and not by the activity of the bacteria; on the contrary, they impede the correct solubilizing activity of *R. aquatilis* AZO16M2, reflected in the low concentrations of soluble phosphorus obtained during the tests (Table 1). Even so, there are other mechanisms carried out by AZO16M2 such as the synthesis of siderophores (Table 2), which capture phosphorus from Fe–P compounds in response to iron stress [49,65,78], favoring the release of available phosphorus (organic or inorganic) [48], reported in several bacteria [74,75,79,80]. Likewise, *R. aquatilis* AZO16M2 synthesizes acid and alkaline phosphatases (Table 2), enzymes involved in the phosphate mineralization process [81] with effects on plant growth [57]. Additionally, their presence is based on the use of the substrate p-nitrophenyl phosphate [82]. Several studies have reported the presence of phosphatase genes [83] in PGPR capable of synthetizing acid and alkaline phosphatases as main mechanisms in phosphorus release [46,72]. In addition, *R. aquatilis* AZO16M2 has the ability to synthetize IAA, an important phytohormone in plant growth and development [24,55], which promotes root development and better plant establishment [84].

When sand and vermiculite were used as substrate in a 1:1 (*v*/*v*) ratio, there was a significant interaction between the factor bacteria (*R. aquatilis* AZO16M2), phosphorus source and substrate, with a better response in the application of rock phosphate on the variables: aerial fresh weight, aerial dry weight and root dry weight (Table 4), superior to the control by 64.15%, 60.53% and 43.48%, considering that the RP contains between 20% and 40% of P_2_O_5_, a characteristic that makes it more accessible to the plant [85]. Another variable is the chlorophyll content (42.38 SPAD) (Table 4), which corresponds to the foliar Mg% (1.11%) (Table 5). Mg is a fundamental part of the chlorophyll molecule, activator of carbohydrate, fat and protein metabolism, as well as an interventor in the transport of phosphates [86]. In contrast, Ca_3_(PO_4_)_2_ treatments did not outperform the established controls, including the K_2_HPO_4_ control, which is a more accessible source of phosphorus for the plant [85]. This behavior could be due to the intervention of root exudates, a weak cation exchange at the root level or due to the characteristics of the substrate. Singh et al. [87] argue that cation exchange is effected in sandy and young soils, which lead to low absorption of cations, especially calcium and magnesium. This behavior corresponds to the leaf content of macronutrients (Table 5) whose percentage of Mg, Ca, K, N and P did not exceed the controls established in the trial. It is worth mentioning that *R. aquatilis* AZO16M2 synthesizes of AIA favors root proliferation [69,70], promotes its development and influences markedly in plant survival in the presence of Ca_3_(PO_4_)_2_ (Table 8) despite having obtained values very close to the controls.

Similarly, a marked interaction was determined between the factors substrate (Premix N°8), bacteria (*R. aquatilis* AZO16M2) and phosphorus source. When rock phosphate was used, 8.91% longer roots were obtained, with 35.58% and 18.76% more aerial and root fresh weight compared to the control as well as a higher chlorophyll content (94.45 SPAD) (Table 6). While in the presence of Ca_3_(PO_4_)_2,_ only the root fresh weight variable exceeded the control by 14.15%, as did the chlorophyll content (45.45 SPAD) (Table 6). The fresh weight gained in the presence of RP and Ca_3_(PO_4_)_2_ is due to the need for low pH to favor its solubilization [2,23], a condition stimulated by the organic nature of Premix N°8, as well as by the capacity of AZO16M2 to synthesize organic acids [68,71]. In addition, the substrate favors the hydration of the seedlings and improves the formation of floccules that allow the conservation of humidity and retention of nutrients, generating an advantageous environment for the establishment of symbiosis between AZO16M2 and the seedlings. All this favors a better response of *M. acuminata* seedlings to the ex vitro adaptation period, overcoming structural, physiological and anatomical anomalies [88] typical of in vitro maintenance, leading to higher seedling survival observed in the presence of Ca_3_(PO_4_)_2_ (Table 8). The low survival percentages with rock phosphate would be due to its low solubility, circumstances that may vary according to the igneous source of the rock phosphate where it comes from [89] and the interaction between organic matter concentration and calcium (Ca) linked to mass substitution [90], which would explain the low survival of *M. acuminata* in ex vitro conditions with RP on Premix N°8 substrate. This condition corresponds to the foliar Ca concentration of the treatments with rock phosphate (Table 7).

## 5. Conclusions

Semiquantitative and quantitative characterization under in vitro conditions, determined that *R. aquatilis* AZO16M2 solubilizes phosphates due to its ability to synthesize siderophores, acid and alkaline phosphatases, and organic acids such as oxalic acid, 2-ketogluconic acid and D-gluconic acid, synthesized on different pH and temperature conditions. In addition, it was confirmed the presence of the PQQ gene in *R. aquatilis* involved in the synthesis of organic acids.

When comparing the ex vitro establishment test of *M. acuminata* in both substrates, it was observed that the increase in dry matter in sand:vermiculite was higher than in the phosphorus source. Thus, AZO16M2 in the presence of RF had a positive impact on chlorophyll content and aerial and root dry weight. On the other hand, the substrate Premix N°8 showed a clear tendency to increase fresh matter, depending on the phosphorus source used, with root length and chlorophyll content being the variables that were most noticeable in the presence of RF, and root dry weight being most noticeable when the source was Ca_3_(PO_4_)_2_. This interaction between the bacteria, the substrate and the phosphorus source influenced the survival percentage, which shows a better adaptation of AZO16M2 in Premix N°8 with Ca_3_(PO_4_)_2,_ with a survival percentage of 87% compared to 37 % in the control.

For the future, it is necessary to deepen the study of microbial consortia with different plant-growth-promoting capacities in association with phosphate-solubilizing bacteria, particularly with *R. aquatilis* AZO16M2, in ex vitro conditions to know their feasibility as co-inoculants and future inoculants for commercial use in open-field banana crops.

## Figures and Tables

**Table 1:** Quantification of available phosphorus in liquid medium with different phosphate sources.

	NBRIP–AlPO_4_			NBRIP–FePO_4_			NBRIP–Ca_3_(PO_4_)_2_		
Strain	Population (ufc/mL)	pH	mg/L P	Population (ufc/mL)	pH	mg/L P	Population (ufc/mL)	pH	mg/L P
AZO16M2	33 × 10^3^	3.48	2.4	33 × 10^6^	3.27	1.4	47 × 10^10^	4.4	29.6

**Table 2:** Synthesis of AIA, siderophores and alkaline and acid phosphatases of *R. aquatilis* AZO16M2.

Strain	AIA (ppm)	Siderophores	Alkaline Phosphatase (µg pNP/mL/min)	Acid Phosphatase (µg pNP/mL/min)
AZO16M2	33.90	(+)	2.56	2.59

**Table 3:** Content of organic acids synthesized by *R. aquatilis* AZO16M2 at different temperatures and pH.

Temperature (C°)	pH	Oxalic Acid ^1^ (µg/mL)		SD ^2^	2-Keto-Gluconic ^1^ Acid (µg/mL)		SD ^2^	D-Gluconic ^1^ Acid (µg/mL)		SD ^2^
4	5.50	6.5373	ab	0.00802	4.3873	c	0.26034	13.8660	i	0.03012
	6.80	6.5313	ab	0.01850	1.5193	b	0.03900	13.8683	i	0.12920
	8.50	6.5397	ab	0.00902	0.0000	a	0.00000	13.7850	hi	0.01229
6	5.50	6.5320	ab	0.00985	6.0627	d	0.03573	13.7517	hi	0.06621
	6.80	6.5277	ab	0.00929	8.6717	e	0.10340	13.6753	h	0.01986
	8.50	6.5377	ab	0.00723	0.0000	a	0.00000	11.4910	g	0.02081
28	5.50	6.5680	b	0.00458	5.5910	cd	0.08266	9.3400	e	0.02443
	6.80	7.0137	d	0.01250	8.3220	e	0.08800	7.3273	a	0.00902
	8.50	6.6770	c	0.03995	5.6997	cd	0.29612	7.5240	b	0.12450
35	5.50	6.5380	ab	0.01652	6.3663	d	0.03623	10.0033	f	0.00723
	6.80	6.4887	a	0.00902	8.0493	e	2.67808	8.9277	c	0.02532
	8.50	6.5790	b	0.08055	5.9113	d	0.60680	9.2110	d	0.12010

^1^ Concentrations of organic acids with distinct letters are significantly different, according to Duncan’s test (α < 0.05). Confidence limit 95%. ^2^ Standard deviation.

**Table 4:** Factorial means of the response of *M. acuminate* var. Valery in association with phosphate-solubilizing PGPR bacteria, *R. aquatilis* AZO16M2, in sand:vermiculite substrate.

Strain	Phosphorus Source	Number of Leaves ^1^ (cm)		Plant Length ^1^ (cm)		Chlorophyll Content ^1^ (SPAD)		Aerial Fresh Weight ^1^ (g)		Aerial Dry Weight ^1^ (g)		Root Dry Weight ^1^ (g)	
AZO16M2	Ca_3_(PO_4_)_2_	7.8	b	13.9	b	55.28	a	8.89	b	0.83	b	1.33	b
	rock phosphate	6.6	a	13.4	a	42.38	a	3.18	a	0.38	a	0.23	a
^2^ B0	Ca_3_(PO_4_)_2_	8.2	b	16.6	b	54.00	a	12.06	b	1.15	b	1.46	b
	K_2_HPO_4_	8.4	c	19.7	c	48.67	a	16.49	cb	1.38	cb	1.45	c
	^3^ P0	5.6	a	8.4	a	44.59	a	1.04	a	0.13	a	0.18	a
	rock phosphate	5.2	a	8.4	a	41.65	a	1.14	a	0.15	a	0.13	a

^1^ Values with distinct letters are significantly different, according to Duncan’s test (α < 0.05). Confidence limit 95%. ^2^ Treatment without bacteria. ^3^ Treatment without phosphorus source.

**Table 5:** Macronutrient content of the leaves of *M. acuminata* var. Valery under ex vitro conditions in association with phosphate-solubilizing PGPR bacteria, *R. aquatilis* AZO16M2, in sand:vermiculite substrate.

Strain	Phosphorus Source	N (%)	P (%)	K (%)	Ca (%)	Mg (%)
Control ^1^ B0	K_2_HPO_4_	2.69	0.42	6.00	0.70	0.69
Control ^1^ B0	Ca_3_(PO_4_)_2_	3.53	0.21	4.91	0.83	0.85
Control ^1^ B0	rock phosphate	3.53	0.10	3.90	0.83	0.82
Control ^1^ B0	^2^ P0	2.83	0.12	5.70	0.60	0.81
AZO16M2	Ca_3_(PO_4_)_2_	3.16	0.15	5.23	0.73	0.63
AZO16M2	rock phosphate	4.00	0.11	6.20	0.69	1.11

^1^ Treatment without bacteria. ^2^ Treatment without phosphorus source.

**Table 6:** Factorial means of the response of *M. acuminata* var. Valery in association with phosphate-solubilizing PGPR bacteria, *R. aquatilis* AZO16M2, on Premix N°8 substrate.

Strain	Phosphorus Source	Root Length ^1^ (cm)		Aerial Fresh Weight ^1^ (g)		Root Fresh Weight ^1^ (g)		Chlorophyll Content ^1^ (SPAD)	
AZO16M2	Ca_3_(PO_4_)_2_	39.30	b	20.23	b	35.68	bc	45.45	a
	rock phosphate	25.80	b	18.91	b	23.98	bc	94.45	a
^2^ B0	Ca_3_(PO_4_)_2_	25.56	b	20.12	b	30.63	bc	38.16	a
	K_2_HPO_4_	21.74	a	8.97	a	8.65	a	35.39	a
	^3^ P0	27.20	ab	20.99	b	34.86	c	44.18	a
	rock phosphate	23.50	b	12.15	b	19.48	b	40.04	a

^1^ Values with distinct letters are significantly different, according to Duncan’s test (α < 0.05). Confidence limit 95%. ^2^ Treatment without bacteria. ^3^ Treatment without phosphorus source.

**Table 7:** Macronutrient content of the leaf part of *M. acuminata* var. Valery under ex vitro conditions in association with phosphate-solubilizing PGPR bacteria, *R. aquatilis* AZO16M2, in Premix N°8 substrate.

Strain	Phosphorus Source	N (%)	P (%)	K (%)	Ca (%)	Mg (%)
Control ^1^ B0	K_2_HPO_4_	2.16	0.42	4.86	0.73	0.74
Control ^1^ B0	Ca_3_(PO_4_)_2_	2.46	0.42	5.69	0.85	0.52
Control ^1^ B0	rock phosphate	2.32	0.40	2.75	0.80	0.73
Control ^1^ B0	^2^ P0	2.04	0.40	4.15	2.15	0.50
AZO16M2	Ca_3_(PO_4_)_2_	2.27	0.41	5.79	0.60	0.65
AZO16M2	rock phosphate	2.10	0.39	6.75	1.01	0.78

^1^ Treatment without bacteria. ^2^ Treatment without phosphorus source.

**Table 8:** Survival percentage of *M. acuminata* var. Valery under ex vitro conditions.

Strain	Phosphorus Source	Survival (%) Sand:Vermiculite	Survival (%) Premix N°8
Control ^1^ B0	K_2_HPO_4_	75	50
Control ^1^ B0	Ca_3_(PO_4_)_2_	62.5	37.5
Control ^1^ B0	rock phosphate	50	50
Control ^1^ B0	^2^ P0	37.5	37
AZO16M2	Ca_3_(PO_4_)_2_	75	87
AZO16M2	rock phosphate	25	25

^1^ Treatment without bacteria. ^2^ Treatment without phosphorus source.

## Data Availability

Not applicable.
